# Acquiring infection: the challenges of collecting epidemics and pandemics, past, present and future

**DOI:** 10.1098/rsfs.2021.0030

**Published:** 2021-10-12

**Authors:** S. Emmens, N. McEnroe

**Affiliations:** Science Museum, London, UK

**Keywords:** acquiring, infections, challenges, collectings, epidemics, pandemics

## Abstract

Curators of the history of medicine are now facing one of the greatest challenges of their careers—how to collect and preserve objects that convey the impacts of COVID-19 on science, medicine and wider society, while that same pandemic rages around them. But in such a rapidly changing situation, might looking back help us in going forward? Using the world-famous Medicine collections at London's Science Museum, we will explore how both presences and absences in the museum record can shed light on the challenges and dynamics of collecting around pandemic and infectious diseases, as well as the broader field of public health. Most strikingly, we examine why the 1918–19 Spanish flu pandemic left little material culture behind, in sharp contrast with other epidemic diseases. In recent years, so-called ‘rapid response’ collecting has become a tool used frequently by museums to capture ongoing developments and current research. To collect COVID-19 presents unique challenges, both practical and ethical, not least because to collect with a view to posterity is an action that remains highly subjective and is dependent on the decisions and personal interests of the curator as well as the influence of major external events.

## 1. Absences in the museum record

One of the foremost traditional roles of museums is to collect, then catalogue, preserve and interpret for posterity. This is by no means a straightforward process and it is inevitably influenced by both the attitudes and beliefs of those doing the collecting and by the challenges and obstacles they can often face at the time. As a result, historic collections are invariably uneven in content. Sometimes physical objects associated with widespread practices or even historic global events may be barely represented or entirely absent from collections otherwise recognized as exemplars in the associated fields, such as disposable items used for disinfecting. Many researchers have surely been crushed by the revelation that there are no surviving artefacts from a key scientific breakthrough or that examples of a once commonplace, throwaway item appear to have all been … thrown away.

Perhaps the window to capture such objects was simply too brief or perhaps they just did not seem important at the time, or maybe one of a whole host of other practical, ethical or even emotional barriers stopped these and countless other items being saved. In recent years, closer interrogations of museum collections are also revealing how objects that might illustrate the lives and experiences of particular groups and communities, to reflect on disability, ethnicity, gender or sexual orientation can also be missing. These and other absences can sometimes be due to the approach taken to collecting and are, of course, not restricted to collections of health and medicine. But perhaps some of these other absences are more easily explained. For example, collections of historic dress traditionally tend to be rich in ‘Sunday best’ and special occasion outfits, as it was these that were kept and treasured. Curator and historian of fashion, Julia Petrov has noted that the ‘earliest museum collections of historical dress rarely included any fashions that might have been worn within living memory’ [1], a situation that would naturally favour the aforementioned ‘kept and treasured’. A well-worn kitchen apron or typical workman's clothes, while once numerically far more common, were less likely to be preserved, so are rarer survivors.

Whatever the reason, things are lost—often for good. One area where absences can be regularly encountered is in the representation of epidemic and pandemic disease in museum collections. Even the Medicine collection at the Science Museum, acknowledged as one of the finest in the world, has significant gaps in the breadth and scope of such areas within its historic collections. In this article, we will explore collecting around these disease phenomena through the lens of the current COVID-19 pandemic, which we led together with our colleagues across the Science Museum Group. We will set out the context for this collecting project and explore the ethical and logistical challenges that we faced and the particular difficulties posed by collecting ephemeral scientific material.

## 2. Medicine at the Science Museum

Almost the entire first floor of the Science Museum building in South Kensington is taken up with five large intersecting galleries dedicated to the story of medical history. Medicine: The Wellcome Galleries, which opened to the public in autumn 2019, explores what we have progressively learned from and about the human body, how different forms of treatment have emerged and changed over time and in what ways public health measures have influenced the health of both individuals and groups within societies. The galleries also explore what could be considered the more cultural side of science—for example, how belief and faith in treatment have direct health outcomes. The galleries contain a series of striking, newly commissioned artworks to help visitors respond to the story of medicine in visual and creative ways. Perhaps most importantly, visual and audio representations of real people allow both medical professionals and patients alike to share their personal stories, allowing visitors to relate such experiences back to their own lives.

Unfortunately, the galleries were only open for four months before the Science Museum took the almost unprecedented step of closing the museum due to the COVID-19 pandemic—the first time the museum had been closed to visitors since the Second World War. Since this closure and the brief periods of being temporarily re-opened, we and the Science Museum's other medical curators have been attempting to collect a wide range of material that will capture the pandemic and its impact for future generations to see and study. In order to help with this challenging collecting, we have also been looking back to discover if conclusions can be drawn from examining the historic collecting around similar areas.

The Medicine collection at the Science Museum comprises the extensive core of Sir Henry Wellcome's historic museum collection alongside the Science Museum's significant later additions. Wellcome (1853–1936) was a pharmaceutical millionaire, born in Minnesota but whose business was based in London where he later became a naturalized British citizen. His passion was for collecting health and medicine across continents and cultures, believing that through the study of such an accumulated mass of objects would come a greater understanding of humanity [2]. His wealth allowed for collecting on an extravagant scale, and at his death in 1936, his collection comprised over 1 million objects. Now largely dispersed, 115 000 core objects primarily relating to the history of medicine were kept by the Wellcome Trust and transferred on long-term loan to the Science Museum in the 1970s [3]. This transfer marked the formal birth of the museum's Medicine collection. The collection now contains well over 150 000 items and is increasing every day as curators actively gather material that we hope will reflect medical developments, breakthroughs, triumphs—and failures—as well as everyday practices and experiences for the use of future research and display.

## 3. The structure of medical museums

Like a number of other major medical collections found elsewhere in the world, the richness and breadth of the historic material found at the Science Museum are undeniable. Why then might collecting around epidemic and pandemic disease events be particularly problematic—as is suggested by the evidence of museum catalogues, not only in the Science Museum but in other museum collections relating to medicine? Here, broadly speaking, we can define an epidemic as a marked prevalence of a disease within a community, population or region and a pandemic when this spreads across entire countries, continents or the whole world. Such events present a striking contrast to the endemic diseases that exist at a background level within such populations all the time. Of course, as conditions shift and circumstances change and bacterial or viral strains adapt—an endemic disease such as seasonal flu could occasionally evolve into an epidemic or even pandemic.

Creating, building and shaping collections of the history of medicine, and with it the simultaneous potential for ‘creating’ absences, is not confined to millionaires or national museums. There, the absence of material might perhaps be ascribed to the influence of connoisseurial preferences or museological elitism. It is also a tradition with deep connections to those who actually practice medicine. The medical profession has a strong affection for its own heritage, and physicians, surgeons and more recently, pharmacists and nurses, are among practitioners who have saved objects to form their own discreet collections. Although varying in scale, stature and scope, this urge to preserve their own heritage is a notable and widespread characteristic of the medical professions. Such collections can be found among the various royal medical colleges and societies and across the hospital trusts. Some were created as a physical and very prominent record of professional prestige, whereas others may owe their more modest existence to the often-unbidden efforts of dedicated volunteers. *Weir's Guide to Medical Museums in Britain* of 1993 lists museums that have medical parts to their collection, as well as ones dedicated entirely to medicine. Although some of the collections listed have now become subsumed into others, it still provides a useful overview of medical collections nationally. From this book, we can see that some areas dominate—‘personalia’ of famous medical professionals, surgery and other instruments, hospital collections with a strong local connection, and specialist collections relating to very specific areas of medicine and a handful of medical military museums [4].

Whatever the origins, when it comes to what goes on display, in the past both amateur collectors and medical curators in museums large and small have tended to over-concentrate on the ‘hero’ objects—what we might now refer to as ‘wow’ objects. This inevitably means that in many areas, representations of the everyday in collections can be severely under-represented in museum displays. But medical museums storerooms tell a different story. Medical collections large and small contain many examples of mundane tools of the trade, especially in the case of surgical collections. Admittedly an extreme example as an especially enthusiastic collector, Henry Wellcome's historic collection contains around 700 pairs of artery forceps, a tool that has fundamentally changed little in its design over the three centuries since it was invented. So if the everyday is carefully kept in collections relating to surgery, anatomy or personalia of famous medical practitioners, why is there so relatively little historic material relating to some of the major epidemics and pandemics of the past?

## 4. The Spanish flu pandemic

Perhaps the best example of a general absence of a pandemic in collections of medical history is the 1918–19 Spanish flu pandemic. Almost certainly the deadliest disease event in history, the total number killed may have been as high a 100 million, with over 500 million infected. Yet Laura Spinney argues it features in the public consciousness purely as a footnote to the First World War. ‘When asked what was the biggest disaster of the twentieth century, almost nobody answers the Spanish flu.’ [5]. The way that Spanish flu almost misses a perceived place in history is perhaps mirrored in historic medicine collections—by the very absence of objects relating to this seismic event.

In the medicine collections at the Science Museum, items clearly associated with the Spanish flu can be almost counted on one hand. They are at best tangential to the pandemic, being examples of over-the-counter medicines from the period and a face mask or two dating from that time, which potentially could have been used. Searching the Science Museum's extensive digital catalogue, the term ‘Spanish flu’—and variations thereof—produce not a single return. Conversely, objects with direct associations with the First World War are numbered in the low thousands. Other museum collections do have more material associated with that pandemic, such as advertisements sharing public health directives, and items relating to preventing the spread of infection such as disinfectant, handwashing and masks. But as a rule, material culture relating to Spanish flu in museum collections is very thin.

The reasons for the lack of museum objects relating to this disease event would appear to be complex and overlapping. The pandemic unfolded very quickly—with the vast majority of deaths occurring in the autumn months of 1918, at the very time that the First World War was coming to a close. Both swift and brutal was the effect on nations exhausted by the conflict. Spinney argues that a natural response to this widespread trauma of death—frequently the death of young and otherwise healthy people—was to avoid thinking and speaking of it. The widespread loss of people in communities who would ordinarily have been the family providers had long-lasting and widespread impacts, especially on children left orphaned. To illustrate one community deeply impacted by the Spanish flu pandemic, she quotes a 1994 report of Yupik people in Alaska. Yupik elder Harold Napoleon writes that the epidemics experienced over two centuries caused such trauma that the only response was silence. ‘To this day *nallunguaq* remains a way of dealing with problems or unpleasant occurrences in Yupik life’, Napoleon writes, ‘Young people are advised by elders to *nallunguarluku*, ‘to pretend it didn't happen.’’ [5]. Although this is a very explicit example of silencing, Spinney suggests that this avoidance of a devastating subject can be seen more widely. Is it a step too far to suggest that this avoidance can be seen only too clearly in the gaps in medical collections relating to the Spanish flu? This could particularly be the case when we consider that for many people, the pandemic was consolidating the trauma already experienced by many during the First World War.

## 5. The risk of losing the ephemeral

Other reasons for the lack of objects representing many epidemic and pandemic events are more practical. Much of the equipment and other material called on in these crises simply carried on being used for ordinary medical needs until they reached the end of their natural life. They were generally neither specialized tools nor new innovations, but just everyday items of nursing, hygiene nor laboratory kit. Another consideration for the lack of material culture is that the items in use might have been (or at least considered to have been) highly infectious and therefore burnt or otherwise disposed of soon after use—often as standard practice to minimize health risks. A housework manual of 1926 gives domestic insight into such an approach, recommending that the housewife, following an infectious disease in the home, completely disinfects the sickroom, ‘… including the clothes of the nurse and of everything used by the patient which cannot be destroyed. Books given to read should be of little value and should be burnt, as they cannot be satisfactorily disinfected.’ [6].

Material relating to major disease outbreaks can also be highly ephemeral; perhaps mass produced for a specific and ultimately time-limited purpose. As with all ephemera, unless steps are taken to preserve examples it will disappear as rapidly as it appeared. During the AIDS pandemic in the 1980s, every household in the UK was posted a public health information leaflet entitled ‘AIDS - Don't Die of Ignorance’. Upwards of 20 million were printed but within a few years, only a handful of these are to be found in museum collections and archives. Other copies undoubtedly survive hidden away in the backs of domestic drawers and cabinets—indeed the one on display in the Science Museum was found squirreled away in a box in a curator's shed—but despite its huge print run, this example largely went the way of all leaflets that come through the front door.

Unlike COVID-19, there was no vaccine for the Spanish flu, so all measures were preventative or concerned with the treatment of symptoms. The use of government directives—encouraging handwashing, discouraging public gatherings and ordering good ventilation—all leave no physical trace, bar the printed communications and messaging. This messaging, as posters and leaflets, along with newspaper advertisements for commercially available ‘cures’, survive in some museum collections, but are not especially numerous.^1^ Even rarer are examples of an item all too familiar to those living a century after the outbreak of the Spanish flu—the face mask. It was once as ubiquitous as it is now in our fight against COVID-19, but now very few survive in museum collections.^2^

## 6. Childhood epidemics, polio and Ebola in the museum

The Spanish flu pandemic is a strong example of a disease that made barely an impression in museum collections despite its enormous impact at the time and for future generations, but it is by no means the only one. Diphtheria, measles and scarlet fever were three of the most common infectious diseases in post-industrial society which together with whooping cough posed the biggest risk to child health and mortality. The impact of these diseases prior to effective vaccination is captured in detail in records kept by Medical Officers of Health at the time, many of which make chilling reading today. The Medical Officer for Islington, reporting on a measles epidemic in 1910 that had killed 160 people, 131 of them under five, wrote: ‘Very large numbers of little ones are daily exposed to unnecessary danger by their attendance at the schools. Indeed, when we look at the roll of deaths from measles and whooping-cough, we cannot, if we have unbiased minds, come to any other conclusion than that the lives of many of the little ones who have died from this disease might have been preserved if they had not been sent to school, where they were brought into contact with the disease^3^’. Yet despite the detail kept in the paper records, the material culture—much of it simple and disposable—associated with these once common epidemics can be sparsely represented in museum collections.

Conversely, there are some epidemics that devastated communities that have fared better in their presence in museums. Polio is one example that clearly caught the attention of collectors and curators, possibly due to innovations in technology that developed as a response to this disease. Rather than a single devastating event, polio emerged to become a recurring seasonal epidemic during the middle decades of the twentieth century. Its regular and appalling reappearances also prompted vaccine research, the successful outputs of which were rolled out across the world from the 1950s. Polio killed but also produced both short-term and lifelong disabilities and over its extended timeframe it developed its own distinct material culture. One such example is a set of four splints used by a Mrs Blackstone, a British woman who was affected by polio as a child in the 1940s. The splints range from heavy to light, as each one was exchanged as her condition improved. As sometimes happens, these splints came to light some 40 years later, when her elderly mother moved to a new house, and cupboards needed clearing. Mrs Blackstone noted ‘The sight of them after so many years was a somewhat disturbing one.’ [7]. Fortunately for us, she donated them to the museum rather than throwing them away. A nice example of a retrospective donation of personal equipment that both shares the experience of the patient and physically records medical treatments of the time.

Like Mrs Blackstone's splints, some of the key technology associated with this particular disease is solid and enduring. The Science Museum is famed for having the largest and best collection of iron lungs in the world—enormous ventilators designed for the patient to lie inside, forcing air in and out of chests paralysed by the effects of polio. The very size of this machinery perhaps contributed to its continuing interest. Expensive to produce, difficult to dispose of and for many years potentially still needed in an emergency, they languished in hospital basements and storerooms until so much time had passed that they had inadvertently gained historic interest. Elsewhere, other less technologically impressive pieces that relate to polio were literally saved from the skip by a curatorial intervention. Among the Science Museum's collections are splints, supports and modified shoes developed for child polio patients at a Hampshire hospital on either side of the Second World War. Poignant artefacts that had endured for decades before fortuitously being retrieved by a visiting curator shortly before the hospital were demolished.

Although we have highlighted the almost total absence of objects in the Science Museum's collections associated with history's deadliest flu pandemic (and for that matter its later, lesser manifestations commonly known as ‘Asian flu’ (1957) and ‘Hong Kong flu’ (1968)), items relating to some other deadly fast-moving, time-limited epidemics have successfully been secured—albeit sometimes unexpectedly. For the Science Museum's curators, collecting material relating to the Ebola epidemic that began in 2014 presented some serious challenges. The outbreaks were happening far away, in Sierra Leone and other regions where it was impossible for us to visit in person. It was also a disease of fearful reputation, with a very high death rate, no effective cure and incredibly infectious. Spread via contact with blood and bodily fluids, 1 cm^3^ of blood has over one billion copies of the virus [8].

Health workers based in infectious areas were subject to a strict regime of safety protocols and protected themselves through layers of personal protective equipment (PPE) which was either destroyed or thoroughly disinfected after each shift. Fortunately, through successful relationship building with, and the significant support of, medical professionals and researchers who volunteered to travel from Britain to West Africa, we were able to acquire a significant cache of objects related to this disease epidemic which would certainly have been otherwise lost. The Ebola material in the Science Museum's collections, much of which is now on public display, provides a unique snapshot of the efforts made against a fast-spreading disease for which there is little defence. With the assistance of the associated medical charities, it was collected in the field and memorably revealed to us in the unlikely surroundings of the Science Museum's café, piece by piece emerging from a large holdall carried back by a Professor of Virology who had just completed his latest tour.

## 7. Collecting COVID-19—a rapid response

So from looking at, as well as partly living through the ongoing history of collecting, today's museum curators have gained an awareness of the challenges of collecting linked to epidemics and pandemics, including in the assessment of what is most at risk of being lost. Like the face masks issued *en masse* in 1918, material that becomes so familiar, so ‘normal’ and is seen everywhere is potentially at risk of being lost due to its very ubiquity. These objects can become almost invisible to those living their ordinary lives during extraordinary times. Many of these items are created in the knowledge they will be rapidly disposed of, perhaps even after a single use. Be it hospital PPE, posters at tube stations, supermarket signage or the crucial but disposable kit used by scientists in laboratories, such things will be naturally or necessarily—or perhaps even joyfully—discarded once they have been used or are no longer needed. But how to apply the insights gained from history when actively attempting to collect the COVID-19 pandemic today?

An approach taken by museums that has gained prominence in recent years has become defined as ‘Rapid Response Collecting’. Objects that appear to reflect current social, economic, scientific or technological change and challenges which prompt debate about associated issues are quickly identified, acquired and in some cases almost immediately put on public display. There is an element of guesswork involved as trying to anticipate now what might become enormously important can be very tricky. More traditional retrospective collecting has the benefit of time, consideration and hindsight, but it does of course carry the inherent risk that the most important—or perhaps the once most ubiquitous—physical remains associated with the subject being addressed have been missed.

The Science Museum is very active in its contemporary collecting. At the time of writing the areas identified for such a proactive response are extremely broad—ranging from microplastics to gaming, and quantum physics to ‘green’ chemistry—to name just a few. As with all collecting, however, the context in which it takes part is crucial. The different circumstances that that dictate the focus of the proactive collecting greatly influence the approach taken and can be the limiting factor in the outcomes. One recent target for collecting by Science Museum curators was a particular climate change protest, from which a number of homemade placards were acquired. They were the specific material culture being sought, but the window of opportunity to gather them was essentially restricted to the day of the protest. Other examples more closely follow that of COVID-19 collecting, where something new emerges, is identified as being of interest then develops over a period of time, during which the choices and opportunities around collecting items are also subject to change and reconsideration.

For the medical curators, the success of such collecting ventures often depends on ensuring that crucial relationships with supportive stakeholders—be they medical professionals, patients or patient advocates—are nurtured and maintained. These colleagues and friends of the museum have the expertise—and the access—that makes contemporary collecting medicine possible. Alongside such expert advice, physical site visits to the places where the things curators are interested in pursuing are actually happening is crucially important. Often sharp-eyed curators can spot material that can seem unimportant, or even irrelevant, to those who work with it every day.

## 8. Ethical challenges in collecting COVID-19

The medical curators at the Science Museum began to collect around COVID-19 during February 2020. It became clear very early on that sourcing and collecting objects and archival material associated with a pandemic, during that very same pandemic, would be less than straightforward. Despite what we had surmised about the gaps in our existing collections, anticipated from our more recent experience of large-scale disease-related acquisitions or through applying the methodologies associated with ‘rapid response collecting’, the challenges have been—and continue to be—significant and they have not always been easily foreseeable.

Ethics were inevitably going to play an important part in this collecting project, so much so that an advisory panel was formed very early on; quite an unusual thing to do for a collecting project. One of the main responsibilities of this panel was to give a steer to the ethical guidelines that needed to be central to this project.^4^ Curators of medical history are no strangers to managing ethical issues—their work can include storing and displaying extremely sensitive material. But the rapid response collecting around COVID-19 threw up an entire new raft of them.

Suddenly, large numbers of existing supporters ranging from NHS staff to biomedical researchers who generously gave their time and expertise had become extremely busy. Similarly, the new relationships that we wanted and needed to forge to pursue objects linked to the many different facets of this pandemic were also proving problematic. Not only was there the issue of how easy would it be to get in touch with them—the main question was, *should* we be distracting people engaged in vaccine research, studying the spread of COVID-19 or working in a hospital that was beginning to experience the pandemic at first hand? Judging when and if to contact people with a view to adding to the Science Museum collection has proved one of the most delicate tasks to manage.

Even when the time was judged right, and scientists and medics have generously met—virtually—with Science Museum curators, it was often the case that objects of interest were still in use. This was especially true when considering cutting edge, and often very expensive, pieces of medical technology. In response, at the present time, in a number of instances, we have requested that items might be donated to the Science Museum when they are no longer needed, quite possibly in many years' time. And it is not only with expensive equipment that curators have had to hold back. When shortages of PPE were clearly acute, the team made the decision to not acquire anything for the collection, no matter how small or seemingly insignificant, that could be of use in protecting life and health. For example, we were kindly offered an example of PPE created using three-dimensional printing in spring 2020, which we refused, preferring it should be used for the reason it was made.

Identifying what *not* to collect is also a challenge in collecting around COVID-19. Purely for practical reasons, the Science Museum decided to largely focus on the British experience of the pandemic. To frame and limit the scope was essential, especially as the range of topics is necessarily broad. One particular area it was decided not to collect was in oral history. The reasons for this decision were threefold—one is that collecting personal experiences of the pandemic is being done very effectively elsewhere, notably by the NHS at 70 project. Second, sourcing, obtaining and processing oral history recordings are very resource intensive and the decision was made, given the limitations on staff movements and access, to focus our energies elsewhere. In addition, ethical considerations again came into play in the decision not to pursue this. The Oral History Society's guidelines around collecting COVID-19-related oral histories warns that ‘when interviewing healthcare or other frontline workers there is a danger that ‘single session interventions that require staff to talk about their thoughts or feelings' may increase the likelihood of PTSD’ [9]. Indeed, the wider emotional cost of attempting to collect material associated with COVID-19, while the pandemic is still playing out, has led the museum community to debate, and in some cases criticise, the rapid response collecting approach.^5^ Another challenge for line managers involved in this collecting project has been to monitor the emotional wellbeing and stress levels of their teams (and themselves), while all working from their own homes. To collect the very thing that is causing your families and communities untold stress is in itself a highly stressful activity.

## 9. Digital COVID-19 collecting

Collecting in challenging circumstances can also highlight issues that were problematic anyway across the museum sector. One of the most significant in regard to the ongoing COVID-19-related collecting concerns digital material. As the pandemic unfolded throughout 2020, it became clear that while much of its associated material culture appeared in recognizable forms that would fit comfortably into traditional collections—labware, signage, posters, vaccine vials and medical tech, etc.—this was also very much a digital event. The digital outputs come from numerous sources and are being produced for many reasons. They also exist in a myriad of different forms and formats. Be it NHS apps or pandemic modelling software, official government tweets or satirical social media GIFs, the volume is overwhelming. Much of it only exists in a digital form, although physical examples of material that has been printed out from a digital source and then used in real settings are being collected when available. For example, this could include posters and leaflets that are produced and made available as digital files to be printed out by the user.

What to collect in the digital sphere is one question that is still being pondered, despite that huge amounts of data having been gathered by curators over the past months. A more fundamental question is how that material might be catalogued, stored and made accessible and what status it might have in comparison with the real, solid and more familiar objects we are also collecting. In addition, much of this material also brings with it issues of copyright and consent that have to be resolved. Finally, while physical objects can inevitably deteriorate over time, digital material has its own set of long-term threats—from file corruption to the likelihood that, like with VHS videotapes, technological advances will mean future access to the original digital formats will become progressively limited [11].

## 10. Collecting COVID-19 vaccine development

At the time of writing, the single topic that preoccupies the world is the rollout of an effective vaccine against COVID-19, in whatever form it might take. The Medicine Galleries already features significant content relating to vaccination. This includes items from other mass vaccination programmes for polio and the successful eradication of smallpox. As it only opened a year ago, there is no mention of COVID-19, so these sections now inevitably lack a certain balance and insight in the wake of the most significant pandemic for a century. A re-display is planned that will incorporate a number of objects acquired as part of the COVID-19 collecting project.

It is sometimes the smallest objects that can pack the biggest emotional punch. The Science Museum has recently acquired and is planning to display the vial that contained the very first vaccine for COVID-19 administered in the mass programme being rolled out across the world. The Pfizer–BioNTech vaccine was delivered to 90-year-old Margaret Keenan in Coventry University Hospital on 8 December 2020, and its container will be displayed alongside the syringe used and the vial that contained her second dose given three weeks later. Nearby will be a further empty vaccine vial—from the very first dose of the Oxford–AstraZeneca vaccine given to Brian Pinker at the beginning of 2021, which has also been acquired by the Science Museum. Without the combined help of NHS England, AstraZeneca, Pfizer, Coventry University Hospital and the Oxford Laboratory, these items would have undoubtedly have been simply placed into a bio-waste bin and lost forever. Now saved, they are two distinctly ‘wow’ objects, but conversely also objects of a type whose normal existence would be fleeting despite being made in numbers over the coming months and years that will run into the hundreds of millions. To continue to collect vaccination development, especially during times when physically visiting places of scientific research will remain a challenge and one that will last for some time to come.

## 11. Conclusion

Writing of the Spanish flu pandemic, Laura Spinney notes that very different factors—from confusion around the contemporaneous medical information made available to the gender-bias of those collecting—can lead to gaps in historic collections: ‘The absences are eloquent in themselves: the lack of a reliable lab test for flu led to massive diagnostic confusion, for example, and women's accounts are relatively rare.’ [12]. How can examining the absences of the past help modern-day curators avoid similar gaps when collecting today? And what can our knowledge of historic collections teach us about collecting now?

To collect with a view to posterity is an action that remains inevitably highly subjective and is dependent on the decisions and personal interests of the curator as well as the influence of major external events. It can also be influenced by the re-evaluation of a museum's existing collections and subsequent shifts in priorities. The specific collection of material relating to public health in the Science Museum has changed in recent years from what was once an inactive collection of ‘hygiene’, represented through soap, combs, bidets and shaving bowls to one of the most important and active collections in the museum. This is in part due to an acknowledgement of the importance of collecting objects, largely overlooked by previous curators, that can represent health and medicine at a population level—a truer reflection of ‘public health’. Such material might reflect on activities that have a direct influence on a population's health—such as the building of a city-wide sewer system. It also includes discreet objects, such as leaflets and posters, that may once also have fallen outside traditional notions around the material culture of medicine.

An understanding of previous approaches, particularly to collecting—or indeed not collecting—around epidemics and pandemics coupled with an awareness of the challenges that such a project might encounter in the current situation, should ideally forewarn and forearm. But we curators need also to be aware of the associated dangers that might be characterized as self-righteous hindsight and ongoing complacency. The attitudes and beliefs, unconscious or otherwise, that current curators bring to this and similar projects may of course one day be criticized by our professional descendants—‘They collected loads of those other things but they didn't get even a single one of these!—What were they thinking!?’

Of course, even when you know what object you want and try to acquire one, it quite often gets away. One small example to illustrate; in January 2020 large, laminated information posters began appearing in UK airports. Through the use of text and simple graphics, it announced the detection of a new infection and asked if those recently arriving from the city of Wuhan in China were experiencing a fever, difficulty breathing or a cough. We tried to get a copy, ideally one to be put aside when no longer needed. It probably seemed an odd request at the time. Shortly afterwards the rapid global spread of COVID-19 rendered such posters obsolete and their public moment passed. Perhaps one will emerge from a store cupboard in the coming months or years, but most likely this early public warning about what was about to become a world-changing event can be considered a new absence in our ever-growing collections.

## Endnotes

1 As examples, poster held at the Temple University Libraries collections, Philadelphia, Pennsylvania, USA, can be seen here: https://digital.library.temple.edu/digital/collection/p16002coll9/id/3157/rec/1 and leaflet from the Collections of the Postal Museum, London, UK, is here: https://www.postalmuseum.org/wp-content/uploads/2020/03/POST_30_4290_image1.8-rotated.jpg.

2 Example at the Rosenberg Library, Galveston, Texas, USA, can be viewed here: https://rosenberg-library.org/wp-content/uploads/2020/04/1918-face-mask.jpg.

3 https://wellcomelibrary.org/moh/report/b18248615/62#?c=0&m=0&s=0&cv=62.

4 Ethical Guidelines – Collecting COVID-19 https://www.sciencemuseumgroup.org.uk/wp-content/uploads/2020/04/SMG-Ethical-guidelines-Covid-19.pdf.

5 For example [10].

## Data accessibility

This article has no additional data.
